# *Kalanchoe pinnata* (Lam.) Pers. Leaf ethanolic extract exerts selective anticancer activity through ROS-induced apoptotic cell death in human cancer cell lines

**DOI:** 10.1186/s12906-024-04570-7

**Published:** 2024-07-15

**Authors:** Nicolas Faundes-Gandolfo, Carlos Jara-Gutiérrez, Mario Párraga, Iván Montenegro, Waleska Vera, Marcela Escobar, Alejandro Madrid, Manuel Valenzuela-Valderrama, Joan Villena

**Affiliations:** 1 Centro Interdisciplinario de Investigación Biomédica e Ingeniería para la Salud (MEDING), Escuela de Medicina, Facultad de Medicina, Universidad de Valparaíso, Valparaíso, Chile; 2 Centro Interdisciplinario de Investigación Biomédica e Ingeniería para la Salud (MEDING), Escuela de Kinesiología, Facultad de Medicina, Universidad de Valparaíso, Valparaíso, Chile; 3 Centro Interdisciplinario de Investigación Biomédica e Ingeniería para la Salud (MEDING), Escuela de Obstetricia, Facultad de Medicina, Universidad de Valparaíso, Valparaíso, Chile; 4 Laboratorio de Química de Metabolitos Bioactivos, Escuela de Química y Farmacia, Facultad de Farmacia, Centro de Investigación Farmacopea Chilena, Universidad de Valparaíso, Escuela de Química y Farmacia, Universidad de Valparaíso, Valparaíso, Chile; 5 Laboratorio de Productos Naturales y Síntesis Orgánica, Departamento de Química, Facultad de Ciencias Naturales y Exactas, Universidad de Playa Ancha, Valparaíso, Chile; 6 Laboratorio de Microbiología Celular, Facultad de Medicina y Ciencias de la Salud, Universidad Central de Chile, Santiago, Chile

**Keywords:** *Kalanchoe pinnata*, Cancer, Antioxidant, Ethanolic extract, ROS, Apoptosis, Chromatin condensation

## Abstract

**Background:**

The leaves of *Kalanchoe pinnata* (Lam.) Pers. (*K. pinnata*), a succulent plant native to tropical regions, are used as a medicinal alternative against cancer in several countries worldwide; however, its therapeutic potential to fight cancer has been little addressed. In this study, we analyzed the phytochemical content, antioxidant capacity, and selectivity of *K. pinnata* leaf ethanolic extract against different human cancer cell lines in vitro.

**Methodology:**

This study subjected the ethanolic extract to enzymatic assays to quantify the phytochemical content (phenolics, flavonoids, and anthraquinones) and its radical scavenging and iron-reducing capacities. Also, the phytoconstituents and major phenolic compounds present in the extract’s subfractions were identified by GC-MS, HPLC, and NMR. Human cancer (MCF-7, PC-3, HT-29) and normal colon (CoN) cell lines were treated with different concentrations of *K. pinnata* leaf ethanolic extract, and the changes in cell proliferation (sulforhodamine B assay), caspases activity (FITC-VAD-FMK reporter), mitochondrial membrane potential (MMP, rhodamine 123 assay), chromatin condensation/fragmentation (Hoechst 33342 stain), and ROS generation (DCFH_2_ probe assay) were assessed.

**Results:**

The results showed that the *K. pinnata* leaf ethanolic extract is rich in phytoconstituents with therapeutic potential, including phenols (quercetin and kaempferol), flavonoids, fatty acid esters (34.6% of the total composition), 1- triacontanol and sterols (ergosterol and stigmasterol, 15.4% of the total composition); however, it presents a poor content of antioxidant molecules (IC_50_ = 27.6 mg/mL for H_2_O_2_ scavenging activity vs. 2.86 mg/mL in the case of Trolox). Notably, the extract inhibited cell proliferation and reduced MMP in all human cell lines tested but showed selectivity for HT-29 colon cancer cells compared to CoN normal cells (SI = 8.4). Furthermore, ROS generation, caspase activity, and chromatin condensation/fragmentation were augmented significantly in cancer-derived cell lines, indicating a selective cytotoxic effect.

**Conclusion:**

These findings reveal that the *K. pinnata* leaf ethanolic extract contains several bioactive molecules with therapeutic potential, capable of displaying selective cytotoxicity in different human cancer cell lines.

## Background

Cancer is a significant public health problem worldwide and is the leading cause of death in more and less economically developed countries [1]. Developing selective chemotherapeutic therapies that improve efficacy and reduce side effects is essential in cancer therapeutics [2]. Activation of apoptotic cell death is a common target of most primary treatments [3]. ROS can induce apoptotic cell death by promoting the loss of mitochondrial membrane potential with subsequent activation of the caspase-dependent apoptotic pathway [4–6]. Accordingly, numerous studies have illustrated that some anticancer compounds of natural origin can induce apoptosis in cancer cells via ROS generation [5, 7–10]. Medicinal plants have been of interest for centuries in cancer treatment, mainly because many currently used drugs with anticancer activity have been obtained from natural resources. Moreover, in recent years, the use of plants and herbs as a source of medical applications has grown, with approximately 30–50% of current pharmaceuticals and nutraceuticals being plant-derived [11]. The genus Kalanchoe (syn. Bryophyllum) comprises approximately 125 perennial succulent species native to Madagascar [12]. The genus has an intricate taxonomy, with a variable number of species and numerous synonyms. Meanwhile, many have been introduced in other areas, sometimes becoming invasive [13]. Furthermore, people have widely spread various species of the genus Kalanchoe, whose leaves are popularly eaten as a remedy against cancer [14]. Studies based on the extracts from some of these species have indicated that they exert cytotoxic activity on tumor-derived cell lines from cervix, breast, lung, liver, colon, and leukemia malignancies [15–18]. Accordingly, members of the Kalanchoe genus produce several active compounds, including flavonoids, steroids, glycosides, bufadienolides, and organic acids, that are responsible for their biological activity [15, 17, 19–21]. *Kalanchoe pinnata* (Lam.) Pers. (*K. pinnata*) is a short member because it grows up to approximately 1.5 m and is commonly used in traditional medicine of tropical regions (Africa, Asia, and Latin America), where the plants grow spontaneously [13]. Leaves and stems taste bitter and, due to their astringent effects, are effective against diarrhea, flatulence, and vomiting [22]. They are used in different countries to treat several diseases and afflictions, such as hypertension and kidney and urinary disorders [23], cough and asthma [24], and also for the treatment of wounds, burns, ulcers, diarrhea, and insect bites [25]. Leaf preparations are used as an antipyretic and to treat malaria in Africa, Asia, and Latin America [26]. Furthermore, extracts from *K. pinnata* display antioxidant and anti-inflammatory activities [27–30]. An in vitro study tested different fractions of *K. pinnata* extracts rich in steroidal glycosides, alkaloids, and steroids and demonstrated concentration-dependent inhibition of human cervical cancer cell proliferation [18]. In a related study, the butanol-soluble fraction from an ethanolic extract of fresh *K. tubiflora* plants produced antiproliferative activity in several cancer cell lines by affecting the mitotic apparatus [31]. A more recent report indicated that the water-soluble fraction of the same extract caused cell cycle arrest and induced senescence in lung cancer A549 cells. Also, this fraction reduced tumor growth in nude mice [15]. Additionally, the ethyl acetate extract of *K. flammea* exhibited selective cytotoxic activity against prostate cancer cell lines, characterized by an augmented ROS production and caspase 3/9 activation [32]. A similar study showed that the aqueous and alcoholic extracts of the *K. thrysiflora* and *K. marmorata* leaves and subfractions (methylene chloride, ethyl acetate, and n-butanol) were selective against the breast cancer cell line MCF-7 when compared with its normal counterpart, HFB4 cells [33]. Despite the abundant information mentioned above, little is known about the selectivity of the *K. pinnata* leaf ethanolic extract.

In this work, we studied the phytochemical composition of the *K. pinnata* leaf ethanolic extract using several analytical techniques (enzymatic, HPLC, and GC-MS), its antioxidant capacity, and the cytotoxic effect of the extract on different human cancer lines by evaluating several relevant parameters, such as proliferation, mitochondrial membrane potential, ROS generation, caspases activation, and chromatin condensation. Our results showed that *K. pinnata* leaf extract is rich in molecules with therapeutic potential, which accounts for its selectivity against human cancer cells by vía ROS production and caspase-mediated cell death. Together, these data expand our knowledge about the antitumoral potential of *K. pinnata* ethanolic leaf extract.

## Materials and methods

### Extraction

*K. pinnata* was collected from Laguna Verde (Latitude: -33.1; Longitude: -71.6833) at 450 m.a.s.l. during January. Kalanchoe specimens are cataloged as common species, and their extraction and usage were performed following Chilean legislation law 20,283 “about native forest recovery and forestry foment” (Ley 20,283 “Ley sobre recuperación del bosque nativo y fomento forestal”) and Decree 28 “rules about resources destined to native forest research” of the Ministry of Agriculture of Chile. Patricio Novoa, Forest Engineer, Botanical Expert, and Chief of the Horticulture Department, National Botanic Garden of Viña del Mar, Valparaíso, Chile, identified all collected plants, considering the plant’s morphological properties. Vouchers for a specimen Kalanchoe pinnata JBN_4536 are kept at the National Botanic Garden of Viña del Mar, Valparaíso, Chile. Extraction procedure: The extraction of aerial parts from Kalanchoe specimens was performed using a non-lethal procedure. Selective pruning was done using the ANASAC PASTA PODA TPN-50 product and fungicide paint for pruning, and it involved different types of wounds on the plant.

Fresh leaves (1.5 kg) were washed with water. Then, the leaf material was air-dried for two days. Extracts were obtained by macerating a fixed amount of dried material with ethanol by soaking the dried powdered plant (500 g) in a bottle with 2 L of ethanol and keeping it for 72 h. The ethanol mixture was then filtered and concentrated by evaporating the alcohol under reduced pressure using a rotary evaporator.

### Phytochemical screening of ethanolic extract

The ethanolic extract was subjected to quantitative chemical tests using standard procedures to identify various bioactive chemical constituents in the leaves.

#### Determination of total phenolic compounds (TPCs)

As previously described, the Folin-Ciocalteu reagent was used to determine the leaf extracts’ total phenolic content (TPC) [34]. Gallic acid was used as a reference standard (20–100 µg/mL) for plotting the calibration curve. The TPCs were determined using a linear regression equation obtained from the standard plot of gallic acid. The TPCs were calculated as means + S.D. (*n* = 3) and expressed as mg of gallic acid equivalent (GAE mM)/g of dry extract.

#### Estimation of total flavonoid content (TFC)

The TFC in the ethanolic extract was determined, as reported by Arvouet-Grand [35]. The amount of flavonoid was calculated from a linear regression equation obtained from the quercetin calibration (Q.E. mM).

#### Total anthraquinone content (TAC)

The TAC in the ethanolic extract was determined, as reported by Mellado et al. [36]. Emodin was used as a standard to construct the calibration curve. The total anthraquinone content was expressed as µg of emodin equivalents (E.E. mM) in the dry extract.

### Antioxidant assays

#### ABTS• scavenging activity

This assay was performed as described by Romay et al. [37]. The total antioxidant capacity of extracts was expressed in mM Trolox^™^ equivalents (TEAC mM) using a standard curve.

#### DPPH radical scavenging assay

The DPPH assay was performed as previously described (Brand-Williams et al. [38]. The disappearance of DPPH• was detected spectrophotometrically at 517 nm. The percentage of RSC (Radical Scavenging Capacity) was calculated using the following equation:

1 $$\text{RSC}(\%)=100\%\times(\text{Ab}_{517}\;\text{control}-\text{Ab}_{517}\;\text{sample})/\text{Ab}_{517}\;\text{control}$$

From the obtained RSC (%) values, the IC_50_ (the concentrations of the ethanolic extract that caused 50% inhibition) was determined by linear regression analysis.

#### Hydrogen peroxide scavenging activity

The ability of leaf extracts to scavenge hydrogen peroxide was evaluated as previously reported [33]. The ability to scavenge the hydrogen peroxide was calculated using the following equation:

2 $$\begin{aligned} &\text{H}_{2}\text{O}_{2}\;\text{inhibition}(\%)=100\%\times\\&\quad[(\text{Ab}_{230}\;\text{control}-\text{Ab}_{230}\;\text{sample})/\text{Ab}_{230}\;\text{control}]\end{aligned}$$

From the obtained inhibition (%) values, the IC_50_ value was determined by linear regression analysis. All measurements were replicated three times.

#### Ferric reducing antioxidant potential (FRAP) assay

The ferric-reducing power of leaf extracts was determined using the FRAP assay, as previously described [39], with minor modifications. FRAP values were expressed as mM Trolox™ (TEAC mM).

### Chromatographic analysis

#### Gas chromatography

The *n*-hexane and dichloromethane (DCM) subfractions of ethanolic extract were analyzed by gas chromatography (G.C.) coupled to mass spectrometry using a Shimadzu GCMS-QP2010 Plus combination (Shimadzu, Kyoto, Japan) equipped with a fused silica RTX-5 capillary column (30 m x 0.25 mm id, 0.25 μm film; Restek, Bellefonte, PA, USA) at the Farmacopea Chilena, U.V., Chile. The G.C. was operated in splitless mode (30 s sampling time and one µL of sample) with helium as the carrier gas (1 mL min-1 flow) and an injector temperature of 200 °C. The oven and the column were programmed from 50 °C (2 min hold) to 280 °C at a rate of 8 °C min-1 (15 min hold). The mass spectrum was acquired by electronic impact at 70 eV with a mass range of 35 to 500 m/z in full mode scan (1.56 scan s-1). Compounds in the chromatograms were identified by comparing their mass spectra with those in the NIST/EPA/NIH Mass Spectral Library [40–42]. Chromatographic peaks were considered “unknown” when their similarity index (MATCH) and reverse similarity index (RMATCH) were less than 850 and were discarded through this identification process [43, 44]. These parameters refer to the degree to which the target spectrum matches the standard spectrum in the NIST Library (the value 1000 indicates a perfect fit) and compare their retention index with those reported in the literature [42], considering the same type of column or those of commercial standards, when available. The retention indexes were determined under the same operating conditions as a homologous n-alkane series (C10–C30) using the following equation:

3 $$\text{RI}=100\times(\text{n}+\text{Tr}(\text{unknown})-\text{Tr}(\text{n})/\text{Tr}(\text{N})-\text{Tr}(\text{n})),$$

Where n = the number of carbon atoms in the smaller n-alkane; N = the number of carbon atoms in the larger n-alkane; and Tr = the retention time. Component relative concentrations were obtained by peak area normalization.

### HPLC analysis

Polyphenol identification and quantification were performed using a high-performance liquid chromatography method based on that described by Celis-Plá et al. [45], with minor modifications. A methanol/water of ethanolic extract, stored at -80 °C befor**e** analysis, was centrifuged at 4,000 rpm for 5 min and filtered through 0.22 μm before injection into the chromatograph. A high-performance liquid chromatograph with autosampler model SIL20AC, column oven model CTO-20AC, degasser model DGU-20A5, pump model LC-20AD, and diode array detector SPD M20A, Shimadzu, were used. Polyphenol identification was performed by comparison of retention times and absorption spectra of standards (190–800 nm) of phloroglucinol, gallic acid, caffeic acid, rutin, cinnamic acid, quercetin and kaempferol with the chromatographic signals detected in the samples. The retention times of the standards were 4.49, 4.98, 18.91, 25.42, 25.74, 35.00- and 42.12-min. Chromatographic separation was performed on a 20 µL sample volume on an RP18 InertSustain column (4.6 × 250 mm, 5 μm), protected by a Nucleodur C18 Gravity pre-column, in an oven at 30 °C, detecting the analytes at a working wavelength of 280 nm. The mobile phase comprised acetonitrile (A) and 1% phosphoric acid in HPLC water (B). Elution of the analytes was performed in gradient as indicated in the table below. The ethanolic extract was subjected to vacuum liquid chromatography by gradient elution of H_2_O-MeOH to afford 20 fractions (A-T). Fraction D (3.4 g) was further subjected to column chromatography using a mixture of n-hexane-EtOAc (10:0–6:1) as eluting solvents to afford seven subfractions (D1- D7). Fraction D5 (150 mg) was subjected to flash column chromatography on silica gel, eluted with CHCl_3_-MeOH (9:1), to give compound 1 (quercetin, 17.0 mg). Fractions D6-D7 were combined (2.2 g) and subjected to silica gel column chromatography using mixture of n-hexane-acetone (10:0–1:1) as eluting solvents to yield four subfractions (D6.a-D6d.) Subfractions D6.d (255 mg) was preparative TLC on silica gel GF254, eluted with CHCl_3_:MeOH (9.5:0.5) to give compound 2 (kaempferol, 22.5 mg).

### NMR analysis

All reagents were purchased from commercial suppliers and used without further purification. Melting points were measured on an SMP3 apparatus (Stuart-Scientific, now Merck KGaA, Darmstadt, Germany) and were uncorrected. Optical rotations were measured with a sodium lamp (λ = 589 nm, D line) on a Perkin Elmer 241 digital polarimeter (Perkin Elmer, Waltham, MA, USA) equipped with 1 dm cells at the temperature indicated in each case. ^1^H-, ^13^C-, ^13^C-DEPT-135, gs 2D HSQC, and gs 2D HMBC NMR spectra were recorded in CDCl_3_ and CD_3_OD solutions. These were referenced to the residual peaks of CHCl_3_ at δ = 7.26 ppm and δ = 77.00 ppm for ^1^H and ^13^C, respectively, and CD_3_OD at δ = 3.30 ppm and δ = 49.00 ppm for ^1^H and ^13^C, on an Advance Neo 400 Digital NMR spectrometer (Bruker, Rheinstetten, Germany) operating at 400.1 MHz for ^1^H and 100.6 MHz for 13 C. The ESI/MS experiment was conducted on a UHPLC Eksigent1 coupled with the M.S. detector ABSciex1 and Triple Quad 4500 model equipment. Thin layer chromatography (TLC) was performed using Merck GF-254 type 60 silica gel. Column chromatography was carried out using Merck type 9385 silica gel. For analytical TLC, silica gel 60 in a 0.25 mm layer was used, and TLC spots were detected by heating after spraying with 10% H_2_SO_4_ in H_2_O.

### Chemicals

Sulforhodamine B (SRB) and Hoechst 33342 were purchased from Sigma-Aldrich (St. Louis, MO) and used without further purification. Fetal bovine serum, penicillin, and streptomycin were obtained from Hyclone (Santiago, Chile) and used as received.

### Cell lines and cell culture

HT-29 cells (colon cancer cell line), PC-3 (prostate cancer cell line), MCF7 (breast cancer cell line), and CCD 841 CoN (human colon epithelial cell line, CoN) were obtained from the American Type Culture Collection, ATCC (Rockville, MD, USA).

All tested cell lines were maintained in a 1:1 mixture of Dulbecco’s modified Eagle’s medium (DMEM) and Ham’s F12 medium, containing 10% heat-inactivated fetal bovine serum (FBS), penicillin (100 U/mL) and streptomycin (100 µg/mL) in a humidified atmosphere with 5% CO_2_ at 37 °C.

### In vitro cytotoxicity screening using the sulforhodamine B assay

Stock cells (HT-29, PC3, MCF-7, and CoN) were incubated at 37 °C under a humidified atmosphere with 5% CO_2_ for 24 h before the assay. The cell suspension was set up at 3,000 cells per well in a 96-well microplate. The dried extract was dissolved in ethanol 100% at 50 mg/mL and diluted with the growth medium to the desired concentrations (0–500 µg/mL). Solvent control cultures were prepared by adding only 1% ethanol. All culture microplates were incubated at 37 °C in a CO_2_ incubator with humidified 5% CO_2_ for 72 h. At the end of drug exposure, cells were fixed with 50% trichloroacetic acid at 4 °C. After washing with deionized water, cells were stained with 0.1% SRB dissolved in 1% acetic acid (50 µL/well) for 30 min and subsequently washed with 1% acetic acid to remove the unbound stain. Protein-bound stain was solubilized with 100 µL of 10 mM unbuffered Tris base, and the cell density was determined using an ELISA fluorescence plate reader at an emission wavelength of 540 nm using the Gen5 1.07 program. The obtained data were expressed as percentages of viable cells versus solvent control, whose viability was considered 100%. Values shown are the means ± S.D. of three independent experiments in triplicate. The software used to calculate the IC_50_ values was SigmaPlot^®^ version 11.0.

### Morphological assessment of cell apoptosis

After 48 h of treatment, nuclear morphology was analyzed in response to different extract concentrations (0 and 50 µg/mL). The fluorescent dye, Hoechst 33342, revealed morphological changes in the chromatin of cells undergoing apoptosis. Human cell lines (HT-29, PC-3, MCF7, and CoN) were cultured on 24-well chamber slides (1 × 10^4^ cells/mL) and exposed to the extract for 48 h. The control group was exposed to 1% ethanol. After adding the Hoechst 33342 solution (1 µM diluted with PBS), cells were incubated in a dark room at room temperature for 30 min. After washing, they were examined under an immunofluorescence microscope at 465 nm (Olympus IX 81 model inverted microscope).

### Determination of mitochondrial permeability by flow cytometry

Rhodamine 123, a cationic voltage-sensitive probe that reversibly accumulates in mitochondria, was used to detect changes in mitochondrial membrane permeability. Cells (HT-29, PC3, MCF-7, and CoN) were incubated with different concentrations of ethanolic extract (25–500 µg/mL) for 48 h, stained with rhodamine 123 (1 µM) in darkness for 1 h at 37 °C. Then, the medium was removed, and cells were washed twice with PBS. Later, the cells were trypsinized and collected by centrifugation (10 min at 1,500 x g). The supernatant was discarded, and the cell pellets were resuspended in PBS and analyzed by flow cytometry using the FL1 filter. Results are expressed as the percentage of cells with intact mitochondrial membrane potential that were stained.

### Measurement of reactive oxygen species production

Briefly, cells (HT-29, PC3, MCF-7, and CoN) were treated with ethanolic extract (0 and 25 µg/mL) for 48 h. Intracellular ROS levels were visualized after incubation with dichloro-dihydro-fluorescein diacetate (DCFH_2_-DA) at a final concentration of 10 µM. Fluorescent dye was added before 30 min at the extract treatment was concluded. After the incubation, cells were washed once in PBS, trypsinized, and centrifuged. The pellet was resuspended in PBS and examined immediately by flow cytometry using the FL1 filter [46]. Results are expressed as the percentage of DCF-positive cells.

### Determination of caspase activation

The activity of caspases was determined by using a fluorescent inhibitor of caspases tagged with fluorescein isothiocyanate, FITC-VAD-FMK. The CaspACE™ FITC-VAD- FMK in situ marker was obtained from Promega. Briefly, cells (HT-29, PC3, MCF-7, and CoN) were treated with the extract (0 and 25 µg/mL) for 48 h. The cells were incubated with CaspACE™ FITC-VAD-FMK in darkness for 20 min at room temperature. Then, the medium was removed, and cells were washed twice with PBS. Exposed cells were collected by trypsinization and centrifugation (10 min at 1,500 x g). The supernatant was discarded, and the cells were resuspended in PBS and analyzed by flow cytometry using the FL1 filter. Results are expressed as the percentage of cells stained with CaspACE™ FITC-VADFMK [47].

### Statistical analysis

Data are reported as mean values ± S.D. Experiments were repeated three times, with triplicate samples for each. Data were analyzed by Prism6^®^ version 6.0d. Statistical significance was defined as *p* < 0.05. After normality tests (Kolmogorov–Smirnov). One-way ANOVA followed by Tukey-Kramer multiple comparisons test as post hoc were applied.

## Results

### Phytochemical content of the *K. pinnata* leaf ethanolic extract

After obtaining the ethanolic extract from *K. pinnata* leaves, the phytochemical content, including total phenolic contents, flavonoids, and anthraquinone, was determined using colorimetric assays. The results are summarized in Table 1.

**Table 1:** Phytoconstituents in the *K. pinnata* leaf ethanolic extract. Total phenolic compounds (TPCs), total flavonoid content (TFC), and total anthraquinone content (TAC) are shown. GAE = gallic acid equivalents, Q.E. = quercetin equivalents, and E.E. = emodin equivalents (means ± S.D., *n* = 3)

				Phytoconstituents					
	TPCs (GAE mM)			TFC (Q.E. mM)			TAC (E.E. mM)		
*K. pinnata* extract	0.020480	±	0.000080	0.005520	±	0.000590	0.017640	±	0.000210

### Total antioxidant activity of the *K. pinnata* leaf ethanolic extract

The antioxidant activity of *K. pinnata* extract was evaluated in a series of in vitro DPPH scavenging, H_2_O_2_ scavenging, and FRAP assays (see Table 2). The *K. pinnata* ethanolic extract showed lower DPPH• radical and H_2_O_2_ scavenging activities but lower ABTS• radical and ferric-reducing antioxidant activity than standard pure compounds such as gallic acid, BHT and TROLOX™ (*p < 0.05).

**Table 2:** Antioxidant activity of the *K. pinnata* leaf ethanolic extract. TEAC = trolox equivalent antioxidant capacity, IC_50_ = inhibitory concentration to scavenge the DPPH or H_2_O_2_ radicals by 50%, and N.A. = does not apply (means ± S.D., *n* = 3, *p* ˂ 0.05)

	**Total antioxidant capacity**											
	ABTS• scavenging activity (TEAC mM)			DPPH• scavenging activity (IC_50_ mg/mL)			H_2_O_2_ scavenging activity (IC_50_ mg/mL)			**Ferric-reducing antioxidant capacity** (TEAC mM)		
*K. pinnata* extract	0.019040*	±	0.000360	24.977410*	±	0.469810	27.619550*	±	1.198590	0.000600*	±	0.000003
Gallic acid	1.139520	±	0.013850	N.A.			N.A.			1.728840	±	0.025820
BHT	1.063020	±	0.027940	0.061210	±	0.000030	2.526760	±	0.056860	1.525940	±	0.076820
Trolox®	N.A.			0.106660	±	0.005770	2.863340	±	0.032140	N.A.		

### GC-MS analysis

The *n*-hexane and dichloromethane (DCM) subfractions of *K. pinnata* leaf ethanolic extract were analyzed by chromatographic analysis (GC/MS). The results of this analysis are shown in Table 3 and Table 4. The *n*-hexane fraction contained high amounts of fatty acid esters, including ethyl palmitate, ethyl linoleate, and 9, 12, 15-octadecatrienoic acid ethyl ester (34.60% of the total extract composition; see Table 3). The DCM fraction had the same fatty acid esters, 1-triacontanol, and the sterols ergosterol and stigmasterol (15.40% of the total composition; see Table 4).

**Table 3:** *n*-hexane subfraction of the *K. pinnata* leaf ethanolic extract. R.T.: retention times; ^a^ RI: retention indexes relative to C10-C30 n-alkanes on the RTX-5 MS capillary column; ^b^ R.I. ref.: retention indexes from the literature [48]; ^c^ surface area of the G.C. peaks; Match: comparison and analysis of the mass spectra with those of the NIST 14; Co: co-elution with standard compounds available in our laboratory [48]

*N*° Peak	*R*.T. (min)	Cas *N*°	Main components	RI^a^	RI ref.^b^	%Area^c^	Match	Reference
1	4.68	2370-12-9	1-pentanol, 2,2-dimethyl	950	-	8.40	87	-
2	5.01	17257-81-7	2-hexanone, 3,4-epoxy	963	-	8.05	88	-
3	22.66	628-97-7	Ethyl palmitate	1999	2000	28.30	96	(Maia et al. 2004)
4	24.02	7541-49-3	Phytol	2080	2096	2.40	93	(Lawal et al. 2009)
5	24.55	544-35-4	Ethyl linoleate	2136	2139	15.63	94	(Babushok et al. 2011)
6	24.64	1191-41-9	9,12,15-Octadecatrienoic acid, ethyl ester	2153	2153	34.60	90	(Tzakou et al. 2006)
7	24.88	111-61-5	Ethyl octadecanoate			2.62	92	(Povolo et al. 2009)

**Table 4:** Dichloromethane subfraction of the *K. pinnata* leaf ethanolic extract. R.T.: retention times; ^a^ RI: retention indexes relative to C10-C30 n-alkanes on the RTX-5 MS capillary column; ^b^ R.I. ref.: retention indexes from the literature [48]; ^c^ surface area of the G.C. peaks; Match: comparison and analysis of the mass spectra with those of the NIST 14; Co: co-elution with standard compounds available in our laboratory [48]

*N*° Peak	*R*.T. (Min)	Cas *N*°	Main Components	RI^a^	RI ref.^b^	%Area^c^	Match	Reference
1	22.60	54546-22-4	Ethyl 9-hexadecenoate	1994	-	0.93	91	-
2	22.67	628-97-7	Ethyl palmitate	2000	1999	19.40	96	(Isidorov et al. 2001)
3	24.03	7541-49-3	Phytol	2080	2096	9.68	97	(Lawal et al. 2009)
4	24.56	544-35-4	Ethyl linoleate	2138	2139	15.25	95	Babushov et al. 2011
5	24.65	1191-41-9	9,12,15-Octadecatrienoic acid, ethyl ester	2156	2153	31.04	96	(Tzakou et al. 2006)
6	24.88	111-61-5	Ethyl octadecanoate	2199	2197	1.83	89	(Povolo et al. 2009)
7	30.58	5908-87-2	Docosanoic acid, ethyl ester	2500	2593	1.26	85	(Andriamaharavo et al. 2014)
8	31.46	593-50-0	1-triacontanol	2554	-	0.70	95	-
9	34.41	57-87-4	Ergosterol	2734	-	2.40	92	
10	35.49	83-48-7	Stigmasterol	2800	3170	13.00	92	(Xu et al. 2012)

### Identification of major phenolic compounds in the *K. pinnata* leaf ethanolic extract

**Fig. 1:**
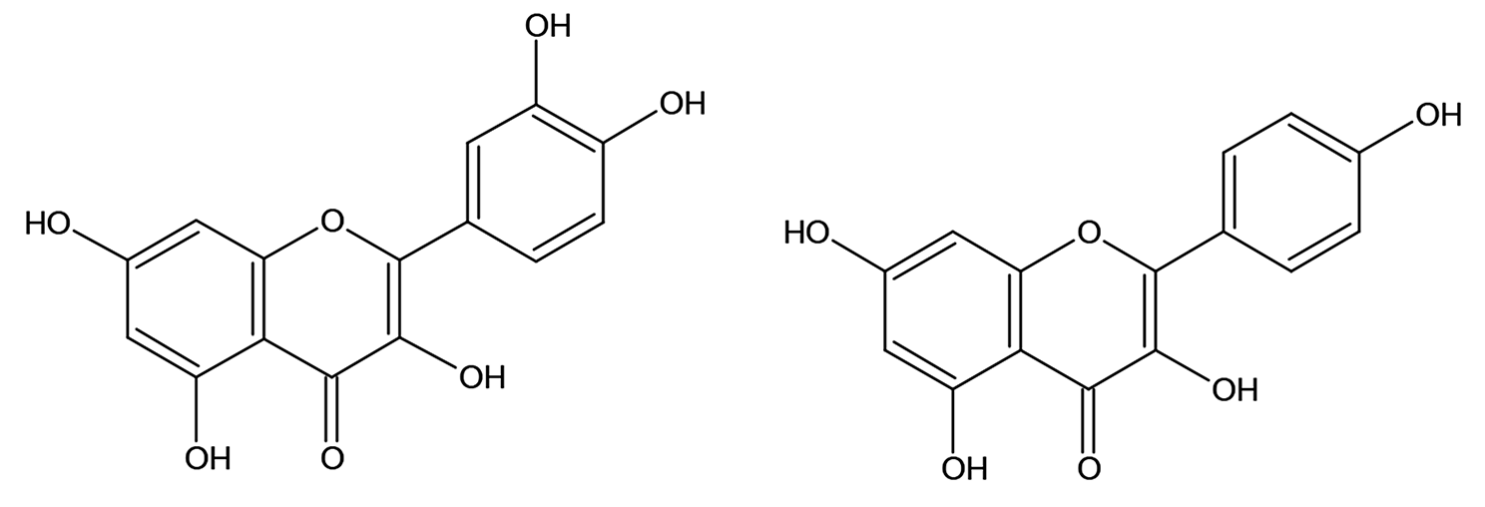
Structure of two major phenolic compounds present in the *K. pinnata* leaf ethanolic extract

Considering that the colorimetric assay revealed the presence of phenolic compounds in the *K. pinnata* leaf ethanolic extract (Table 1), we wanted to identify the major phenolic compounds present in the extract. Thus, two major phenolic compounds (compounds 1 and 2, see Material and Methods section) were isolated by HPLC, and their chemical structure was elucidated by NMR spectroscopy ( Fig. 1). The spectroscopy data of quercetin (obtained from subfraction D5) and kaempferol (obtained from subfraction D6.d) are detailed as follows:

Quercetin: Yellow amorphous powder; U.V. (MeOH):λmax (log ε) 274 (3.8), 360 (3.6) nm; I.R. (KBr) νmax cm^− 1^ 3430, 1680, 1610, 1250; ^1^H-NMR (CD_3_OD, 400 MHz):δH 7.65 (1H, d, J = 2.1 Hz, H-2′), 7.50 (1H, dd, J = 8.4,2.1 Hz, H-6′), 6.85 (1H, d, J = 8.4 Hz, H-5′), 6.40 (1H, d, J = 2.0 Hz, H-8), 6.20 (1H, d, J = 2.0 Hz, H-6); ^13^C-NMR(CD_3_OD, 100 MHz): HRMS (ESI) spectral data(m/z 302.2263 calculated forC_15_H_10_O_7_ m/z 302.40).

Kaempferol: Yellow amorphous powder; U.V. (MeOH): λmax (log ε) 272 (4.0), 364 (3.7) nm; I.R. (KBr)νmax cm^− 1^: 3420, 1690, 1605, 1260, 720; 1 H-NMR (CD_3_OD,400 MHz): δH 8.04 (2 H, dd, J = 11.5, 2.8 Hz, H-2′, H-6′),6.95 (2 H, dd, J = 9.8, 2.7 Hz, H-3′, H-5′), 6.52 (1 H, d,J = 2.0, H-8), 6.28 (1 H, d, J = 2.0, H-6); ^13^C-NMR(CD_3_OD, 100 MHz); HRMS (ESI) spectral data (m/z 285.2263 [M-H]+), calculated for C_15_H_10_O_6_ m/z 286.2270.

### The *K. pinnata* leaf ethanolic extract displays an antiproliferative effect on human cancer cell lines

The antiproliferative effect of the *K. pinnata* leaf ethanolic extract was assayed in human cancer cell lines derived from different cancer types: HT-29 colon cancer, PC-3 prostate cancer; MCF-7 breast cancer; and one non-tumorigenic cell line, CCD 841 CoN (CoN) human colon epithelial cells. The extract was applied using ethanol as a solvent at a final concentration of 1%. The effect of the *K. pinnata* extract on the proliferation of these human cell lines was evaluated using the sulforhodamine B colorimetric assay. The data obtained in response to different extract concentrations (0–500 µg/mL) was used to calculate the IC_50_ and selectivity index (SI) values, as listed in Table 5.

**Table 5:** IC_50_ (µg/mL) and SI values of the *K. pinnata* ethanolic extract for different human cancer cell lines and normal CoN cells

Cell line	IC_50_ (µg/mL)	SI
HT-29	0.20 ± 0.08	8.40
MCF-7	2.68 ± 1.04	0.66
PC-3	14.45 ± 0.54	0.11
CoN	1.68 ± 0.24	n.a.

The data correspond to the mean of the IC_50_ values ± S.D. of three experiments in triplicate. The selectivity index (SI) for the extract in each cell line was calculated as IC_50_ non-tumoral cell line CoN/ IC_50_ cancer cell line. n.a.= not applicable.

The ethanolic extract exhibited an antiproliferative effect on all studied cell lines, including the non-tumorigenic CoN cells, in all tested concentrations. IC_50_ values ranged from 0.2 to 14.4 µg/mL. These values are much lower than those needed to consider plant extracts as active and possible anticancer agents [49]. HT-29 cells exhibited the highest cytotoxicity value, while PC-3 cells showed the lowest. Interestingly, the cytotoxicity of this extract was lower on normal CoN colon cells than on HT-29 cells (SI = 8.4), indicating selectivity in this type of cancer cell line (Table 5). A key feature of an effective anticancer drug is its ability to kill cancer cells selectively, not its ability to kill cancer cells at low concentrations. Thus, selectivity is the most critical feature of an effective anticancer drug [50]. Moreover, Koch et al. [51] suggested that an SI > 2 is an interesting result that suggests considering a compound or extract as a possible candidate for an antitumor drug.

### The *K. pinnata* leaf ethanolic extract affects mitochondrial membrane potential in human cancer cell lines

**Fig. 2:**
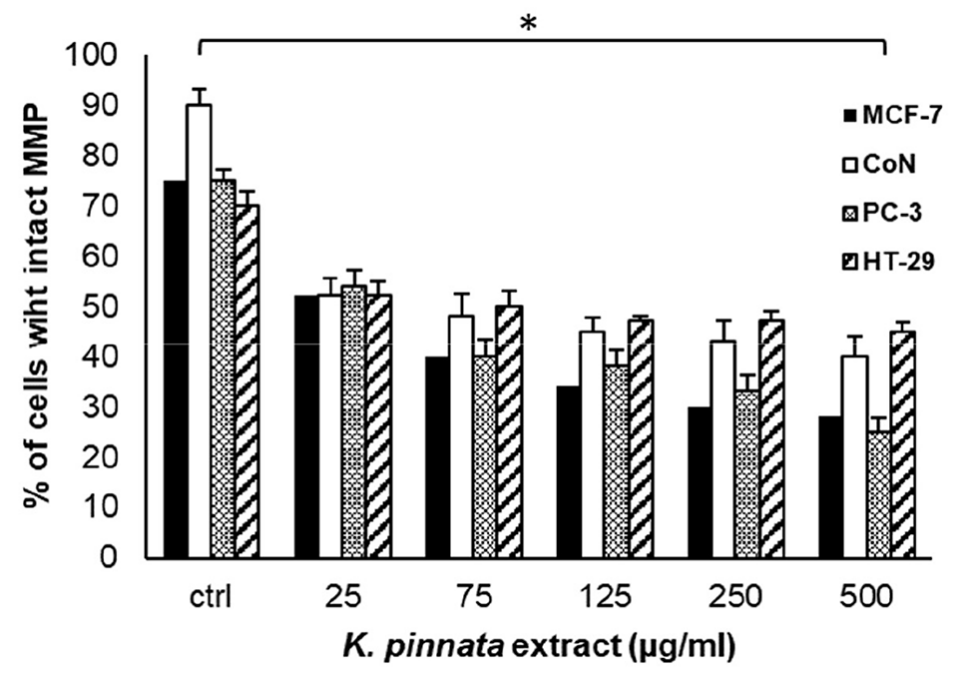
Effect of the *K. pinnata* leaf ethanolic extract on the mitochondrial membrane potential of different cancer-derived and normal CoN cells. Cells were exposed to different extract concentrations (0–500 µg/mL) for 48 h. All data are reported as the percentage of cells (%) with intact mitochondrial membrane permeability compared to control cells (1% ethanol) (means ± S.D., *n* = 3, *p < 0.05)

Since mitochondria play a crucial role in the apoptotic cascade, being a convergent center of apoptotic signals originating from both the extrinsic and intrinsic pathways [52], changes induced in mitochondrial membrane permeability have been reported to exert a significant effect on the execution of cell death. In agreement with this notion, we analyzed the impact of the *K. pinnata* leaf ethanolic extract on mitochondrial membrane potential using flow cytometry with rhodamine 123 staining. The accumulation of rhodamine 123 in mitochondria is associated with an intact electrochemical gradient in mitochondria [53]. As shown in Fig. 2, for all cell lines tested, the percentage of cells with intact mitochondrial membrane potential decreased in response to *K. pinnata* leaf ethanolic treatment (ranging from 25 to 500 µg/mL for 48 h). Loss of mitochondrial membrane potential leads to the release of apoptogenic factors, such as cytochrome c, which is implicated in the activation of caspases [52].

### The ***K. pinnata*** leaf ethanolic extract induces ROS production in human cancer cell lines

**Fig. 3:**
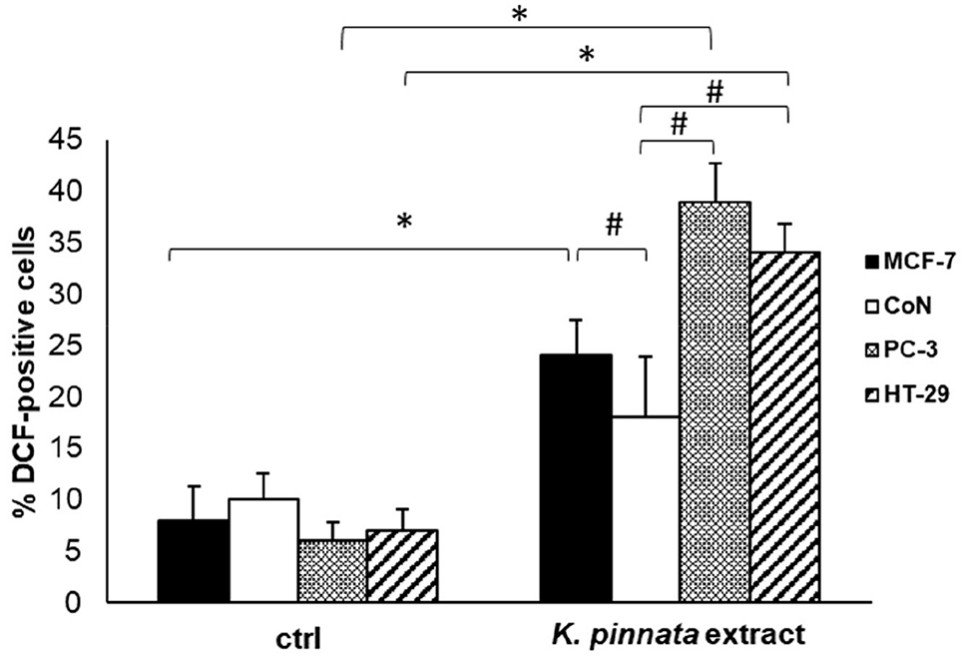
Effect of the *K. pinnata* leaf ethanolic extract on ROS generation. Cells were treated with the extract (25 µg/mL for 48 h), and intracellular ROS levels were determined in three human cancer cell lines (MCF-7, PC-3, and HT-29) and one non-tumorigenic cell line (CoN) by flow cytometry using dichloro-dihydro-fluorescein diacetate (DCFH_2_-DA). * *p* < 0.05 versus control-treated cells (ctrl); # *p* < 0.05 versus ethanolic extract-treated CoN cells. The data represent the means ± S.D. of at least three experiments with triplicate samples

The generation of reactive oxygen species (ROS) and mitochondrial damage are frequently associated with cell death induction. ROS levels were assessed after 48 h of ethanolic extract treatment (25 µg/mL) using the fluorescent probe dichloro-dihydro-fluorescein diacetate (DCFH_2_-DA). DCF fluorescence was then measured by flow cytometry. Changes in the percentage of DCF-positive cells in treated cells versus ethanol-treated cells were interpreted as increased intracellular ROS. The extract caused a statistically significant increase in intracellular ROS in all cell lines tested (Fig. 3), except in CoN cells. Moreover, the increased amount of ROS was statistically significant versus the extract-treated CoN cell line.

### The *K. pinnata* leaf ethanolic extract induces caspase activity in human cancer cell lines

**Fig. 4:**
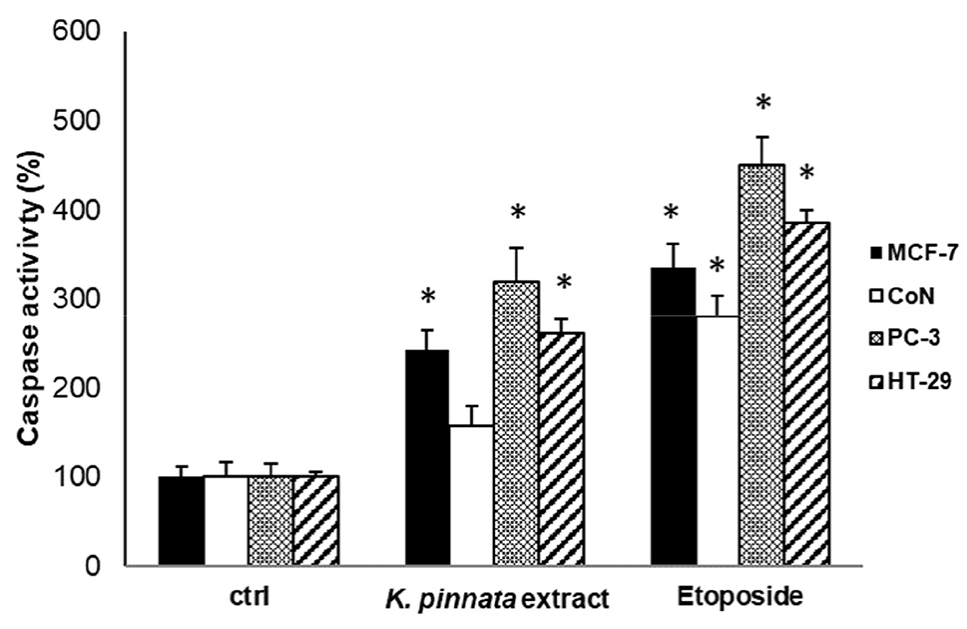
Effect of the *K. pinnata* leaf ethanolic extract on caspase activity in human cancer cell lines. Caspase 3/7 activity was determined in MCF-7, CoN, PC-3, and HT-29 cells following treatment with the *K. pinnata* ethanolic extract (25 µg/mL) for 48 h. Data is reported as the ratio of activity in treated cells vs. those in control cells (1% ethanol), which were arbitrarily assigned a unitary value. Etoposide (25 µM) was included as a positive control. * *p* < 0.05 versus control-treated cells (ctrl). Data are reported as mean values ± S.D. from three different experiments with samples in triplicate

Since ROS levels were increased in the human cancer cell lines in response to the *K. pinnata* extract, we wondered if this effect was associated with the onset of apoptotic cell death. With this in mind, cells were treated with the *K. pinnata* ethanolic extract (25 µg/mL) for 48 h, incubated with the CaspACE™ FITC-VADFMK in situ marker for active caspases, and then analyzed by flow cytometry. As shown in Fig. 4, exposure to *K. pinnata* significantly increased caspase activity in the cancer cell lines but not in CoN cells. As a positive control, cells were treated with the chemotherapeutic drug etoposide, a recognized proapoptotic molecule with poor selectivity. Notably, the increase of caspase activity was quite similar between the positive control and *K. pinnata* ethanolic extract treatments.

### The *K. pinnata* leaf ethanolic extract promotes chromatin condensation in human cancer cell lines

**Fig. 5:**
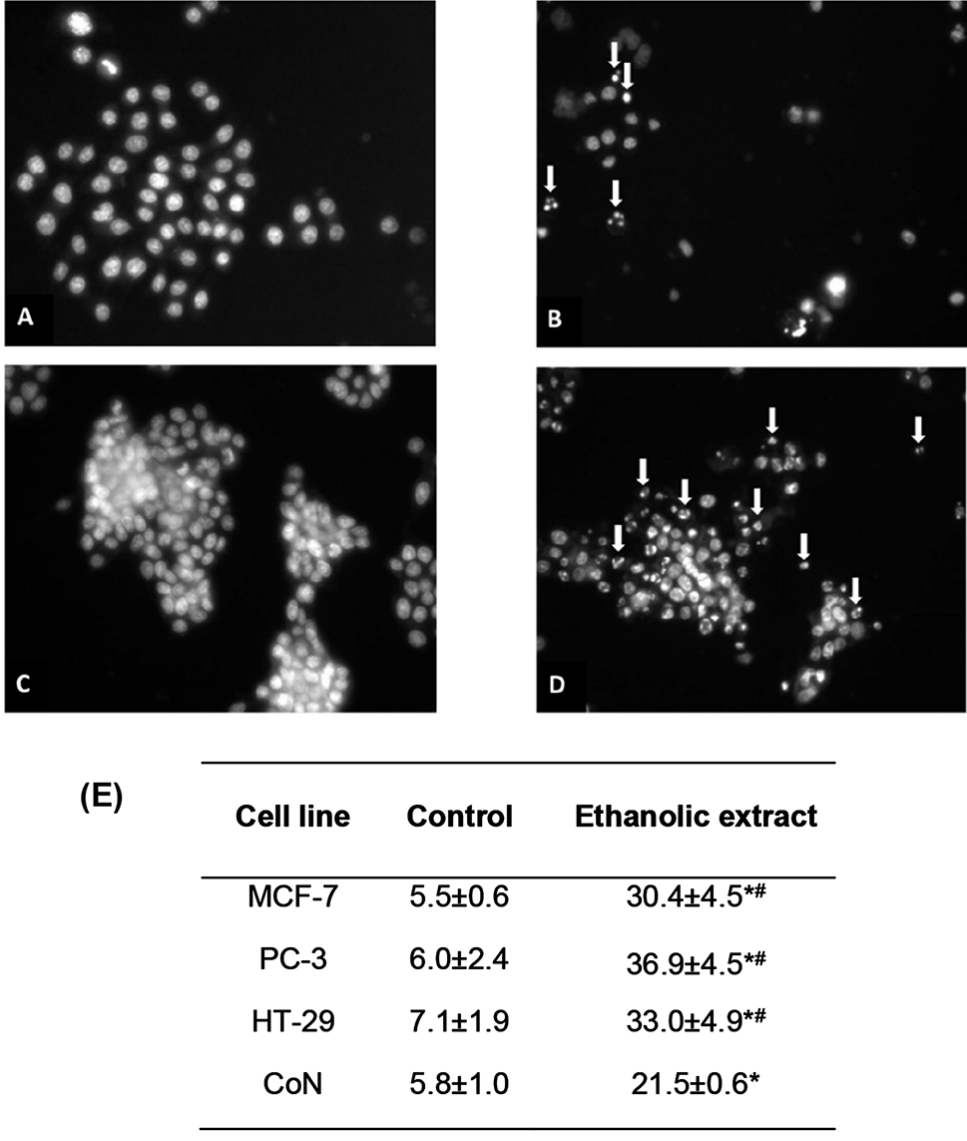
Effect of the *K. pinnata* leaf ethanolic extract on chromatin condensation in human cell lines. (**A**-**D**) Representative photographs of changes in chromatin condensation obtained by fluorescent microscopy. CoN and HT-29 cells were exposed to 1% ethanol as a control condition (**A** and **C**, respectively) or treated with the *K. pinnata* ethanolic extract (50 µg/mL) for 48 h (**B** and **D**, respectively). The arrows indicate condensed/fragmented nuclei. (**E**) Percentage of cells with condensed/fragmented chromatin in control and treated conditions for MCF-7, PC-3, HT-29, and CoN cells (* p < 0.05). Significant differences in chromatin condensation following *K. pinnata* ethanolic extract treatment in cancer cells are indicated compared to CoN control cells in similar conditions (# *p* < 0.05). Data are reported as mean values ± S.D. of three independent experiments

Finally, changes in chromatin condensation following treatment with the ethanolic extract were assessed by Hoechst 33342 staining using fluorescence microscopy. Fig. 5 shows the images (A-D) obtained by fluorescence microscopy (40×) after Hoechst 33342 staining. CoN and HT-29 cells in the control condition (Fig. 5A C, respectively) and treated with the *K. pinnata* leaf ethanolic extract (Fig. 5B and D, respectively) were examined for nuclear morphology changes after 24 h of treatment. The ethanolic extract-treated cells showed a reduction in number, suggesting increased cell death. Moreover, compared to control cells, cells treated with the ethanolic extract showed significant chromatin condensation/fragmentation (Fig. 5B and D, arrows). Cleavage of chromosomal DNA is a biochemical hallmark of apoptosis [54]; thus, this observation agrees with the induction of caspase activity in human cancer cells in response to the *K. pinnata* ethanolic extract (Fig. 3). As shown in the table (Fig. 5E), the percentage of cells with chromatin condensation/fragmentation following treatment with the *K. pinnata* ethanolic extract significantly increased in all the tested cell lines; however, this effect was more pronounced in the cancer cell lines (HT-29, MCF-7, and PC-3) than in the non-tumorigenic cell line CoN.

## Discussion

More than 85–90% of the world’s population relies chiefly on plant-based traditional medicine [55]. Over 3000 plant species are traditionally used worldwide to treat cancer [56]. The genus Kalanchoe comprises approximately 125 species. Various species of this genus, whose leaves are eaten by people, have been popularly spread among cancer patients [57–59]. Different extracts of these Kalanchoe species have been used in traditional medicine, including cancer treatment [57].

An in vitro study demonstrated a concentration-dependent inhibition of human cervical cancer cell growth when tested for *K. pinnata* chloroform extract and a fraction containing steroidal glycosides, alkaloids, and steroids. Results showed IC_50_ values of 552 µg/mL and 91 µg/mL in the crude leaf extracts and fraction, respectively [18], in a similar way as our data show. However, our IC_50_ values are definitively lower than those observed by Mahata and colleagues [18]. It is important to mention that selectivity is an essential feature of an effective anticancer treatment, which is also valid for *K. pinnata* ethanolic extract. Finally, our IC_50_ values are lower than those indicated by Manosroi and colleagues [49] to consider plant extracts as active and possible anticancer agents.

Earlier studies by Yamagishi et al. [60] demonstrated that methanolic extracts of *K. pinnata* exerted cytotoxicity against epithelial K.B. cells. In that study, the IC_50_ value obtained from HPLC-purified active compounds from chloroform extracts ranged between 10 ng/mL-4 µg/mL. These values are similar to those observed in the current study for HT-29 colon cancer cells (0.2 µg/mL). Of note is that our data are related to ethanolic extract and not purified compounds. Additionally, the antitumor activity of *K. pinnata* leaves has also been demonstrated in the context of its antimutagenic activity [61].

Considering that it is possible to find phytoconstituents with antioxidant capacity in plant-derived ethanolic extracts, we performed enzymatic assays to quantify these molecules. The results revealed a reduced concentration of these molecules in the *K. pinnata* leaf ethanolic extract, which was associated with a poor antioxidant capacity. For instance, the extract’s antioxidant capacity was lower than those observed for the antioxidants used as the positive control in TRAP and FRAP assays. On the other hand, the IC_50_ of the extract is higher than the IC_50_ of positive controls in DPPH and H_2_O_2_ assays, indicating lower antioxidant activity than the positive control. When comparing the antioxidant activity of the extract under study with positive controls TROLOX, gallic acid, and butylhydroxytoluene, all show significant differences with *p* < 0.05.

Several anticancer agents currently used for cancer treatment have been shown to cause an increase in cellular ROS generation and are logical candidates for evaluating the strategy of preferentially killing cancer cells with increased ROS stress. Because normal cells appear to have low levels of ROS stress and reserve a higher capacity to cope with further oxidative insults than cancer cells, it is possible to use agents that directly or indirectly cause ROS accumulation to preferentially kill cancer cells and improve therapeutic selectivity [4]. One significant effect is to generate increased intracellular ROS, causing loss of mitochondrial membrane potential and, consequently, apoptosis. The ethanolic extract of *K. pinnata* increased ROS generation in all cell lines except in non-tumorigenic CoN cells, suggesting a selective effect on cancer cells.

Therapeutic agents for cancer include arsenic trioxide, anthracyclines, cisplatin, bleomycin, and irradiation. These previously used anticancer drugs exhibited relatively high toxicity to tumor cells and normal cells [62]. A reasonable way of assessing anticancer activity in vitro would be to test if the drug candidate kills cancer cells without significantly affecting nonmalignant cells [50]. Selectivity is a more relevant feature than cytotoxicity for an anticancer drug. Our data indicates that the *K. pinnata* ethanolic extract has selective cytotoxic activity, at least in HT-29 cells, suggesting it may be a possible anticancer agent.

In related studies, extracts of various species of Kalanchoe (*K. diagremontiana*, *K. pinnata*, *K. milloti*, and *K. nyikae*) were analyzed for cytotoxic activity. The IC_50_ values (µg/mL) ranged from 49.9 to 1,410 µg/mL, indicating a significant variation in the activity of the extracts and cell line-dependent effects on cytotoxicity [63]. Many authors have isolated and identified some chemical constituents of the genus, which can be classified mainly as flavonoid glycosides, anthocyanins, coumarins, bufadienolides, sterols, phenanthrenes, and fatty acids [59]. Indeed, n-hexane and DCM subfraction were analyzed by GC-MS, revealing the presence of sterols, fatty acids, and other compounds with antitumoral activity (Table 3 and Table 4). Our data suggest that the most relevant compounds in the n-hexane and DCM subfraction of *K. pinnata* leaf ethanolic extract are phytol, stigmasterol, ethyl palmitate, and ethyl linoleate. Phytol and its metabolites at concentrations in the physiological range (≤ 10 µM) can alter pathways involved in carcinogenesis, such as increasing apoptosis, decreasing proliferation, and inhibiting the cancer stem cell population [64–66]. Stigmasterol is a relevant phytosterol in various herbal plants and possesses anticancer activity. Studies demonstrate that stigmasterol inhibits endothelial cell proliferation, migration, and capillary network formation [64]. Ethyl palmitate induces apoptosis in HepG2 cells, perturbing the cell cycle [67]. Finally, ethyl linoleate induces apoptosis in human cervical carcinoma HeLa cells through apoptosis, increasing the proteolytic activation of caspase-3 [68].

Moreover, HPLC and NMR analysis identified quercetin and kaempferol as the major phenolic compounds in the *K. pinnata* ethanolic extract. Kaempferol is a flavonoid aglycone found in many natural products [69]. Kaempferol inhibits various types of cancer cells by triggering apoptosis, cell cycle arrest at the G2/M phase, downregulation of phosphoinositide 3-kinase (PI3K)/protein kinase B (AKT) pathway, expression of EMT-related markers and metastasis-related markers [69–72]. On the other hand, quercetin is the principal representative of flavonols. Quercetin is ubiquitously present in different fruits and vegetables [73]. The anticancer effects of quercetin include its ability to reduce the proliferation of cancer cells [74–76], induce apoptosis [77], and cause cell cycle arrest [78] by modulating the activity of critical molecules for cell cycle progression, such as cyclins, PI3K/Akt signaling [79, 80] and mitogen-activated protein kinase (MAPK) pathway [81].

The results of the present investigation demonstrated that *K. pinnata* leaf ethanolic extract contains several bioactive compounds that exert a selective cytotoxic effect on human cancer cell lines, characterized by ROS production, loss of mitochondrial membrane potential, and induction of apoptotic cell death.

## Conclusions

This study shows that the ethanolic extract obtained from *K. pinnata* leaves is rich in molecules with therapeutic potential, such as quercetin and kaempferol, although poor in antioxidant molecules. In particular, the ethanolic extract showed selectivity only when the IC_50_ value obtained in the human colon cancer line HT-29 was compared with its normal counterpart, CoN cells (SI = 8.40). Despite this result, a selective effect was observed when parameters such as ROS generation, caspase activity, and chromatin condensation/fragmentation were compared between normal CoN cells and cancer cell lines (MCF- 7, PC-3, and HT-29), where the extract showed augmented cytotoxicity on the group of cells of malignant origin. These results reveal a selective effect of the *K. pinnata* leaf ethanolic extract against cancer cells, suggesting that this extract could constitute a promising source of antitumor bioactive compounds for pharmaceutical applications.

## Data Availability

Any additional information required to reanalyze the data and reagents reported in this paper is available upon request to the corresponding authors.

## Declarations

### ***K. pinnata*** recollection

*Kalanchoe pinnata* is an introduced plant in Chile, therefore, it is not required a special permit to collect or use the plant in our country. Plant leaves were obtained from a hatchery (Mr. Victor Basilio Añasco Gonzalez, hatchery producer. Los Álamos, Parcela 1, Limache, Región de Valparaíso, Chile. (33° 00’ 58.0’’S – 71° 15’ 52.5’’W). Plant extraction and usage were performed following Chilean legislation law 20,283 about Native Forest Recovery and Forestry Foment and Decree 28 “Rules about resources destined to native forest research” of the Ministry of Agriculture of Chile.

## Abbreviations

K. pinnata: Kalanchoe pinnata (Lam.) Pers
GC-MS: Gas chromatography-mass spectrometry
HPLC: High-performance liquid chromatography
NMR: Nuclear magnetic resonance
FITC-VAD-FMK: Fluoro isothiocyanate conjugate of the cell-permeable caspase inhibitor VAD-FMK
DCFH2-DA: 2’,7’-dichlorodihydrofluorescein diacetate
ROS: Radical oxygen species
GAE: Gallic acid equivalent
TFC: Total flavonoid content
TPCs: Total phenolic compounds
TAC: Total anthraquinone content
ABTS: 2,2’-azino-bis(3-ethylbenzothiazoline-6-sulfonic acid
TEAC: Trolox equivalent antioxidant capacity
DPPH•: 2,2-Diphenyl-1-picrylhydrazyl
RSC: Radical scavenging capacity
IC50: Inhibitory concentration 50%
FRAP: Ferric reducing antioxidant potential
MeOH: Methanol
TLC: Thin layer chromatography
FBS: Fetal bovine serum
PBS: Phosphate buffer saline
PIK3: Phosphoinositide 3-kinase
AKT: Protein kinase B
MAPK: Mitogen-activated protein kinase
